# Variation in the rate of recovery in motor function between the upper and lower limbs in patients with stroke: some proposed hypotheses and their implications for research and practice

**DOI:** 10.3389/fneur.2023.1225924

**Published:** 2023-08-03

**Authors:** Auwal Abdullahi, Thomson W. L. Wong, Shamay S. M. Ng

**Affiliations:** Department of Rehabilitation Sciences, The Hong Kong Polytechnic University, Kowloon, Hong Kong SAR, China

**Keywords:** stroke, upper extremity, lower extremity, motor recovery, natural instinct for walking hypothesis, bipedal locomotion hypothesis, central pattern generators hypothesis, role of spasticity hypothesis

## Abstract

**Background:**

Stroke results in impairment of motor function of both the upper and lower limbs. However, although it is debatable, motor function of the lower limb is believed to recover faster than that of the upper limb. The aim of this paper is to propose some hypotheses to explain the reasons for that, and discuss their implications for research and practice.

**Method:**

We searched PubMED, Web of Science, Scopus, Embase and CENTRAL using the key words, stroke, cerebrovascular accident, upper extremity, lower extremity, and motor recovery for relevant literature.

**Result:**

The search generated a total of 2,551 hits. However, out of this number, 51 duplicates were removed. Following review of the relevant literature, we proposed four hypotheses: natural instinct for walking hypothesis, bipedal locomotion hypothesis, central pattern generators (CPGs) hypothesis and role of spasticity hypothesis on the subject matter.

**Conclusion:**

We opine that, what may eventually account for the difference, is the frequency of use of the affected limb or intensity of the rehabilitation intervention. This is because, from the above hypotheses, the lower limb seems to be used more frequently. When limbs are used frequently, this will result in use-dependent plasticity and eventual recovery. Thus, rehabilitation techniques that involve high repetitive tasks practice such as robotic rehabilitation, Wii gaming and constraint induced movement therapy should be used during upper limb rehabilitation.

## Highlights

- There is reported difference in the rate of recovery of motor function between upper and lower limbs following stroke. The latter is believed to recover faster than the former.
- One of the reasons attributed to this is that, the cortical homunculus of the upper limb is larger in size due to its higher tactile sensitivity.
- We also proposed natural instinct for walking, bipedal locomotion, central pattern generators hypotheses to further help explain the reasons for the difference.
- However, most importantly, the difference could a factor of intensity or frequency of use of the lower limb compared to the upper limb, and spasticity.
- Therefore, interventions for upper limb motor function should consider increasing the intensity and effective management of spasticity.

## 1. Introduction

Stroke causes impairment in motor, sensory and cognitive functions. For the motor function, its impairment results in disability in carrying out activities of daily living (ADL), which can negatively affect the patient’s quality of life (1–4). Thus, for stroke survivours to regain the ability to carry out ADL such as feeding, bathing, wearing clothes, grooming and picking up the telephone to answer calls, recovery of upper limb motor function is needed (5). Similarly, recovery of lower limb motor function is essential for walking which is required for ADLs such as transfer from one place to another, going for shopping and participating in social and other activities (6). In addition, recovery of motor function, independence in carrying out ADL, and the ability to participate in social and other activities are important in achieving good quality of life (7, 8). Therefore, the importance of upper and lower limb motor function recovery cannot be overemphasized.

However, to date, the rate of recovery of upper and lower limb motor function following stroke is a subject of debate that requires the attention of clinician scientists and researchers. For instance, for a very long time, it has been suggested that, the difference is due mainly to the size of the areas representing the limbs in the cortical homunculus. The area representing the upper limb is larger than that of the lower limb (9, 10); and as such, it was suggested that, its recovery may take a longer time following stroke. Although this could be a possible explanation for the difference, a more recent evidence has however not shown any significant correlation between lesion volume or size and motor function (11); suggesting that, other factors may be responsible for the difference in the rate of recovery between the two.

In addition, although, some researchers opined that, there is essentially no difference in the rate of recovery between the two (12, 13); yet, some studies reported lower limb to recover faster than the upper limb (14–22). However, the fast recovery of the lower limb compared to the upper limb, has been observed to be in a subpopulation of patients with anterior circulation infarct (14). Anterior circulation supplies brain areas that are mainly responsible for the motor and sensory functions of the lower limb, and speech production (23). Moreover, it is noteworthy in the study by Paci and colleagues that, all the participants included in the study received rehabilitation (14). During rehabilitation, it was observed that more attention is usually given to the lower limb compared to the upper limb (24). Thus, allocating attention to the limb may result in intensive practice during rehabilitation, which is important for use-dependent plasticity and recovery (25). Therefore, this could be another reason for the difference.

Another reason for the variation could be the type of stroke. This is because, ischemic type of stroke generally shows better functional outcomes compared to the hemorrhagic type (26). This is because, hemorrhagic type of stroke is associated with complications such as expansion of hematoma, increased blood pressure, venous thrombotic events and perihematomal oedema with increased intracranial pressure that can cause further damage to brain cells (27). In addition, other factors such as severity of the impairment and age may be the possible explanation for the difference in rate of recovery (28–30). Furthermore, pattern or rate of recovery that is observed following stroke largely depends on the type of outcome measures used to determine the recovery. The neurophysiological measures of recovery such as the transmagnetic stimulation (TMS), are generally more sensitive than the behavioral measures such as the Fugl-Meyer motor assessment (31). Unfortunately, most studies used the behavioral measures to assess recovery following stroke (15–22).

However, considering that all the above arguments may not be exhaustive on the subject matter, there seems to be many other factors which require further investigation that have not yet been considered in the debate on the variation in the rate of recovery of motor function between the upper and lower limbs following stroke (30). The aim of this paper is to propose several hypotheses for the possible difference in the rate of recovery of motor function between upper and lower limbs following stroke, and their implications for research and practice.

## 2. Literature search

For this purpose, five databases, PubMED, Web of Science, Scopus, Embase and CENTRAL were searched from their inceptions to February, 2023 using the key words, stroke, cerebrovascular accident, upper extremity, lower extremity and motor recovery for relevant literature. The search generated a total of 2,551 hits. However, out of this number, 51 duplicates were removed using Endnote software. Thereafter, relevant articles on recovery of motor function were read, and based on our understanding of the reviewed literature, experience and knowledge of the subject matter, we proposed 4 hypotheses: natural instinct for walking hypothesis, bipedal locomotion hypothesis, central pattern generators (CPGs) hypothesis, and role of spasticity hypothesis on the subject matter to help explain why the difference exists. See Table 1 for the summary of the articles guiding the proposed hypotheses.

**Table 1:** Summary of some of the important articles guiding the proposed hypotheses.

Authors	Type of article	Main points from the article	Hypothesis
O’Mara (32)	Narrative review	The article opines that walking is a natural phenomenon adapted by human being for their social participation	Natural instinct for walking hypothesis
Yen et al. (33)	RCT	Early mobilization involving standing and stepping practices resulted in improved ability to carry out ADL and functional ambulation; and reduced length of hospital stay	Natural instinct for walking hypothesis
Awad et al. (34)	Expert review	The authors argue that, human locomotion involves 3 subtasks, propulsion, limb advancement, and body weight support	Bipedal locomotion hypothesis
Abdullahi et al. (35)	Systematic review and meta-analysis	Performing tasks practice with the affected lower limb, while constraining the unaffected limb helps in improving its function including functional mobility	Bipedal locomotion hypothesis
Ryu and Kuo (36)	Modeling study	Walking which is one of the important functions of the lower limb can be produced by central pattern generators (CPGs) located in the spinal cord even in the absence of control of the higher centers	Central pattern generators hypothesis
Minassian et al. (37)	Narrative review	Walking which is one of the important functions of the lower limb can be produced by central pattern generators (CPGs) located in the spinal cord even in the absence of control of the higher centers	Central pattern generators hypothesis
Katoozian et al. (38)	Observational study	Prevalence of spasticity is usually higher in the upper limb compared to the lower limb following stroke	Role of spasticity hypothesis
Kong et al. (39)	Cross-sectional study	Upper limb dexterity is severely affected by the presence of severe spasticity	Role of spasticity hypothesis

RCT, randomized controlled trial; ADL, activities of daily living.

## 3. The hypotheses

### 3.1. Natural instinct for walking hypothesis

Humans seem to have a natural instinct for wanting to walk no matter what. This can be seen even early in life, where stepping/walking reflex, which is the placement of one foot in front of the other when the soles of feet touch ground, is present at birth (32). Although this reflex disappears at age 6 weeks, it voluntarily reappears at age 8–12 months (32). In addition, humans consider walking as a means to an end; and as such they walk to carry out their ADL such as going for shopping, and participating in social and leisure activities (40).

Moreover, historically, it is believed that, “humans made multiple journeys on foot out of Africa to the Eurasian landmass, and dispersing eventually to the Americas and Asia-Pacific region” (41–43). This seems to suggest that, importance of the lower limbs for all human endeavors is as old as the humans themselves. Consequently, in the event of an injury to the nervous system such as after stroke, the natural instinct of the patient is to want to recover walking ability as soon as possible, to help achieve independence in carrying out ADL as much as possible (44, 45). This is probably because, recovery of lower limb motor function significantly influences health-related quality of life (46). Interestingly, early mobilization following stroke results in early recovery (33). In addition, repetitive steps that are taken during walking can help induce recovery of lower limb motor function through use-dependent plasticity (47, 48).

**Figure 1:**
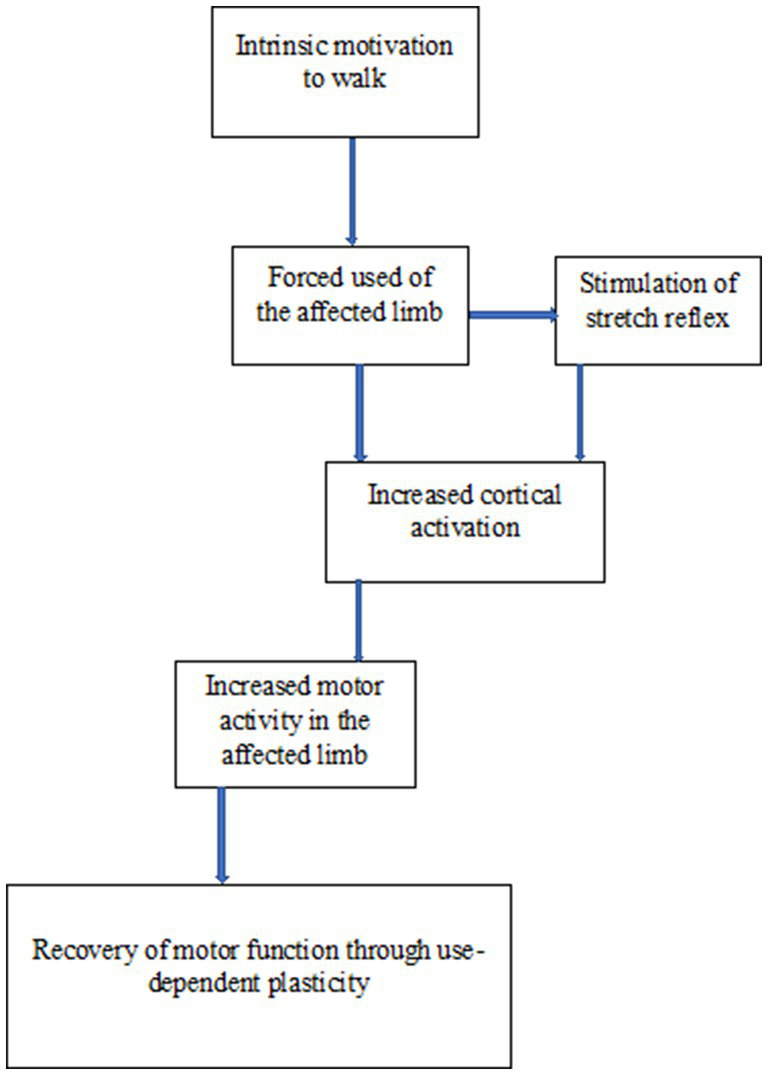
Schematic representation of the mechanism of natural instinct for walking hypothesis.

See Figure 1 for the mechanism of the natural instinct for walking hypothesis.

### 3.2. Bipedal locomotion hypothesis

Human locomotion is bipedal, which involves three subtasks, propulsion, limb advancement and body weight support (34). As such, following stroke, the less affected or sound lower limb can be used during propulsion to help force the use of the affected limb (35, 49–51). Forced use of limb following stroke helps with reversing learned non-use, and promoting recovery (49, 52). In addition, bearing weight on the affected limb that generate proprioceptive information in the foot, can serve as important sources of sensory outputs for recovery (53). Consequently, bearing weight on the affected limb helps with the recovery of walking speed and functional mobility (52, 53).

**Figure 2:**
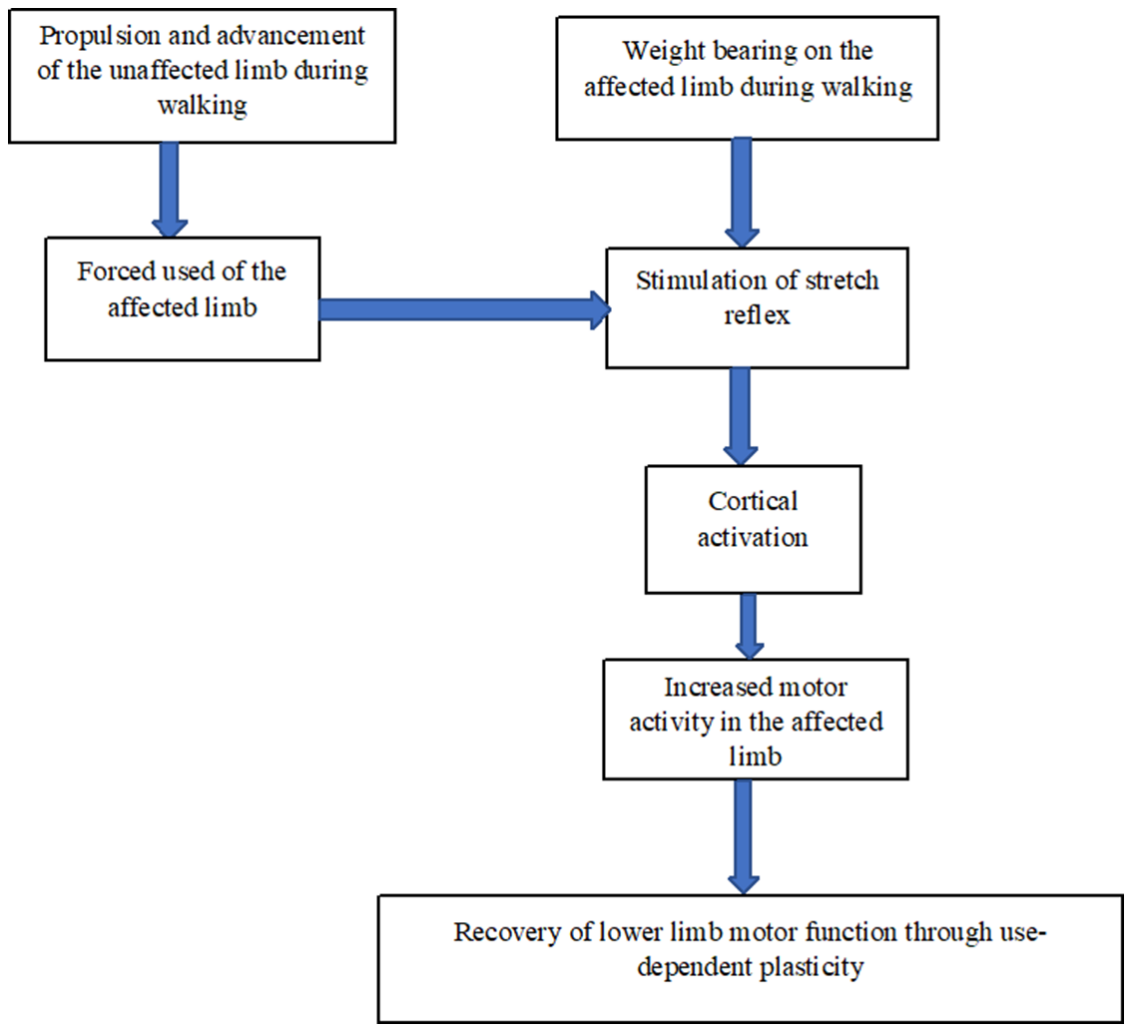
Schematic representation of the mechanism of bipedal locomotion hypothesis.

See Figure 2 for the mechanism of the bipedal locomotion hypothesis.

### 3.3. Central pattern generators hypothesis

Walking in humans is mainly produced by the combined roles of the reflex circuit, which produces motor patterns triggered by sensory feedback, and the central pattern generators (CPGs), which is a network of neurons capable of generating rhythmic pattern movements even in the absence of command from the higher motor centers (36, 37, 54–57). The CPGs innervate mainly the muscles of the lower limb (54); and they may not be affected following stroke. In addition, the neurons that orchestrates walking reside predominantly in the lumbar spine (58, 59). Consequently, rythmic pattern movement such as stepping during walking can be generated even in the absence of control of the higher centers. Evidence of rhythmic-locomotor activity in the lower limb was seen following epidural stimulation of the spinal cord (60).

**Figure 3:**
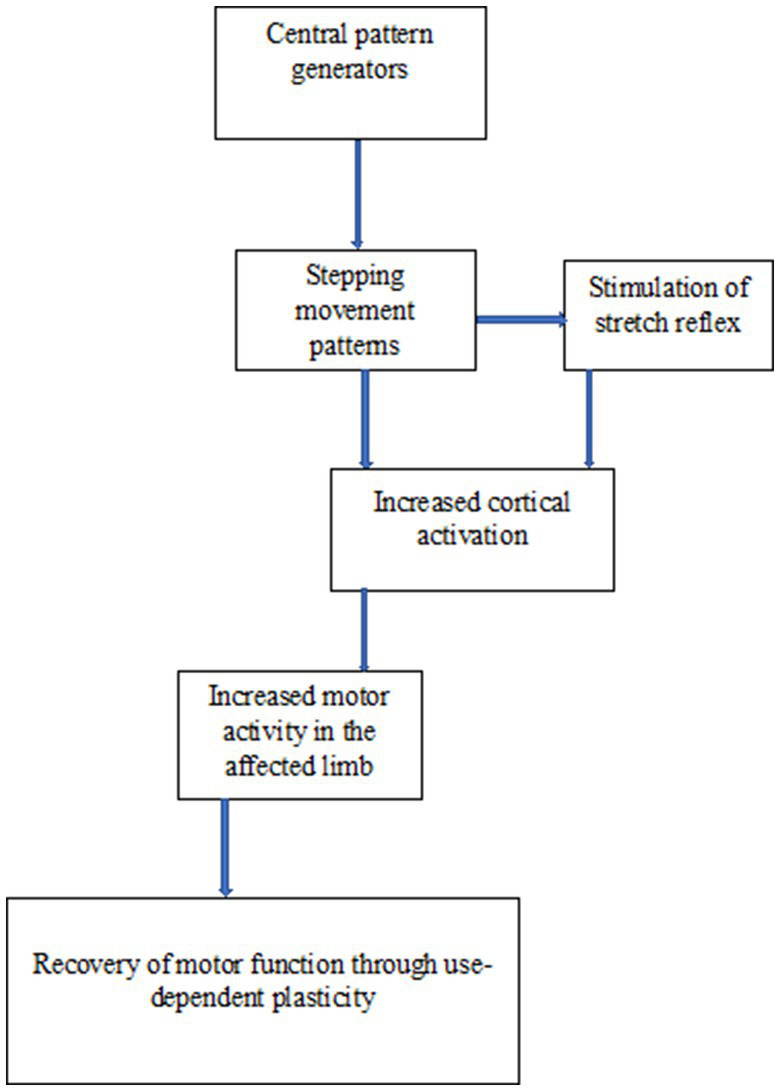
Schematic representation of the mechanism of central pattern generators hypothesis.

See Figure 3 for the mechanism of the central pattern generators hypothesis.

### 3.4. Role of spasticity hypothesis

About 25% of patients with stroke develops spasticity, although it depends on the severity of the paresis (61). However, prevalence of the spasticity and its severity, are higher in the upper limb than in the lower limb (38). Presence of severe spasticity in the upper limb, correlates with poor hand dexterity (39). In addition, unlike the lower limb, spasticity in the upper limb is associated with 60, 100, and 33% cases of shoulder pain, elbow pain and wrist pain, respectively, (62). Presence of pain is a significant predictor of poor recovery of function, ability to carry out ADL and quality of life following stroke (63, 64). In contrast, presence of spasticity may not substantially affect functional recovery of the lower limb (65).

In addition, functional specialization of the upper and lower limbs differs. The upper limb is involved in the performance of complex fine motor movement (66). However, as noted earlier, spasticity in the upper limb is significantly associated with poor dexterity, a requirement for fine motor movement ability (39, 65). Moreover, spasticity is associated with decreased joint proprioception (67). Acuity of proprioception in the wrist joint is linked to the control of fine movement (66). Thus, this may be the reason why even in the presence of motor and functional recovery, use of the upper limb in daily activities, which is also an indicator of recovery, may not be easily achieved (68).

## 4. Discussion, and implications for research and practice

Recovery of motor function following stroke has been considered to depend on so many factors such as the size and location of the lesion, and time since stroke (28–30). Similarly, although it is still debatable, the recovery is considered faster in the lower than the upper limb (14–22). However, following review of the literature, we hereby proposed some hypotheses to help explain other possible reasons why the lower limb may recover faster than the upper limb, and discussed the implications of the hypotheses for research and practice. The hypotheses are natural instinct for walking, bipedal locomotion, central pattern generators and role of spasticity hypotheses.

Following stroke, natural instinct for walking, which will result in motor activity with the affected limb; bipedal locomotion, in which weight is borne on the affected limb, while the unaffected limb is used to propel the affected one; role of CPGs in producing rhythmic movement patterns such as the steps needed during walking; and the role of spasticity in impairing movement, suggest that, the lower limb may recover faster than the upper limb because it is used more than the latter in activities. This is because inadequate amount of activity as may often be the case with upper limb compared to the lower limb, may not be able to drive neural reorganization that is required for recovery (69). Interestingly, walking is an ADL, and use of the limb for daily activities in the real world, is a significant predictor of recovery of motor function following stroke (70).

The above argument seems to suggest that, use-dependent plasticity may be the reason for faster recovery of motor function in the lower limb compared to the upper limb. Thus, increasing activity or intensity of practice during upper limb rehabilitation is important to help optimize recovery, by inducing biochemical, physiological and anatomical changes in the brain (71–74). Increasing the intensity of practice of the affected upper limb can be achieved through the use of technology driven rehabilitation interventions such as the Wii gaming and robotic rehabilitation (75, 76). In addition, techniques such as the constraint induced movement therapy, which comprises of massed tasks practice with the affected limb, constraint of the unaffected limb, and transfer package (a contract to ensure continuous use of the affected at home) should be considered (77–79). Already, it is known that, repetitive tasks practice of the upper limb results in greater recovery (80–82); and this will in turn result in increased use of the limb in the real world (83).

In addition, the larger muscles of the lower limb are very important in maintaining standing posture (84). Thus, because of patients’ natural instinct for wanting to regain walking, they would have to be able to stand first before they can walk. In doing so, bearing weight on the two limbs will automatically stimulate the stretch reflex, which will in turn activate the motor cortex (85, 86). When the motor cortex is repeatedly activated, recovery of motor function ensues (87). In addition, even during walking, weight is continuously borne on the lower limbs which helps with the restoration of motor function through the mechanisms already mentioned above. This is because, control of gait and posture are intricately related (86). Moreover, due to the bipedal nature of human locomotion, the unaffected limb forces the affected one into activity during propulsion and limb advancement. Thus, this can result in use-dependent plasticity, and eventual recovery of the lower limb (49).

Similarly, the role the CPGs play in the generation of rhythmic movement pattern such as the steps required for walking, may aid with the faster recovery of the lower limb (37, 54). Thus, considering the roles play by bipedal locomotion in humans, where the unaffected limb forces the affected limb into activity during propulsion and limb advancement; and the potential role of the CPGs in lower limb recovery, use of rhythmic bilateral movement training and bilateral upper limb exercise may help promote recovery of upper limb motor function through use-dependent plasticity (88, 89). Furthermore, as noted earlier, presence of spasticity in the upper limb is associated with poor recovery outcomes (39). Thus, this seems to suggest that, presence of spasticity may account for the difference in the rate of recovery between the upper limb and the lower limb. As such, managing spasticity in the upper limb during early post stroke may help hasten its recovery. Consequently, effective interventions for spasticity in patients with stroke such as active exercises, joint positioning and joint stretching should be used (90).

Although, the 4 theories proposed in this paper tried to explain some of the reasons why the lower limb motor function recovers faster than that of the upper limb, they are not in any way exhaustive, and as such other factors should also be considered. One of these factors is the argument that, upper limb occupies a larger area in the motor homunculus due its high tactile sensitivity, compared to the lower limb (9). Thus, to help recruit more areas of the brain to aid with the recovery of upper limb motor function, sensorimotor stimulation techniques such as the brain and peripheral electrical stimulation and tactile stimulation can be used in combination with other interventions (91–93). Stimulation of the nervous system can result in recovery of the upper limb (91, 94).

Secondly, the timing of rehabilitation is also important. This is because early post stroke is the period when the potential for recovery is higher (8, 95, 96). In addition, it is important also to note that, ability to determine or predict recovery depends on the outcome measure used (97). Furthermore, the difference sometimes may also depend on the psychometric properties of the outcome measures used (20). For instance, most studies use measures of daily function or disability rather than measures of impairment (20). Thus, in determining and predicting recovery of motor function after stroke, a combination of clinical, neurophysiological and imaging outcome measures should be used (20, 98, 99). Moreover, research is needed to be carried out, where practice/ activity will be controlled between upper and lower limbs, to determine if one will recover faster than the other. Similarly, studies should compare patients with the same degree of spasticity in the upper and the lower limbs to determine which one recovers faster.

## 5. Conclusion

The lower limb may regain motor function following stroke at a rate faster than the upper limb. Although many factors can help explain the reason why, most importantly the reason majorly has to do with the intensity or frequency or dose of use of the lower limb compared to the upper limb, and presence of spasticity and its significant impact on the upper limb. Therefore, rehabilitation strategies for upper limb motor function following stroke should consider increasing the intensity of practice especially in the real world, and management of spasticity, especially during early post stroke.

## 6. Expert opinion

In our opinion, the hypotheses we presented are some of the factors that make the lower limb to recover its motor function faster than the upper limb; and that all of them seem to suggest that, the main factor for the difference is intensity of use of the lower limb compared to the upper limb. However, these factors we hypothesized seem not to be yet thoroughly investigated, and as such, future studies should focus on investigating them. For instance, views or opinions of stroke survivours using qualitative research methodology should be collected to explore what they prefer to recover immediately after having a stroke. In addition, ethnography method of qualitative research, whereby a group’s behavior is observed by the researcher without interfering with their behavior, can be used to observe stroke survivours through their recovery journey. That way, the researchers can document the journeys of recovery of upper and lower limbs motor function with the goal of observing which one of them recovers faster.

Similarly, observational studies using objective outcome measures of motor function (physical function) such as the Fugl Meyer motor assessment and Wolf motor function test (WMFT) can also be used to objectively determine the difference over a long period of at least 1 year. In addition, electrophysiological measures of motor function such as the electromyography (EMG) to measure muscle electrical activity, and functional magnetic resonance imaging (fMRI) to measure cortical activity should also be used to determine the difference. Furthermore, biomechanical measurements of aspects of motor function such as movement speed, smoothness, quality and directness should also be considered. Thus, in determining the difference in recovery of motor function between the upper and lower limbs, a combination of outcomes measures of physical function, electrophysiological function, biomechanics, perspectives or views of patients and the caregivers and participants observation should be used to help with more reliable comparison. Moreover, many variables such the participants’ age, sex, time since stroke, side affected, lesion volume, type of stroke, presence of neglect, and handedness before stroke need to be controlled in the studies.

In addition, in practice, clinicians should consider methods and techniques that will help increase the intensity of practice with the upper limb. For instance, transfer package whereby a contract is designed between the clinicians, the patients and their caregivers to make patients practice with the affected limb more in the real world, particularly at home; and home programs to increase the intensity of practice can be used. Furthermore, self-management techniques such as the use of motivational interviewing that will help increase patients’ self-efficacy to enable them practice more with the affected limb should be incorporated in upper limb rehabilitation. Similarly, use of mechanical and computer devices such as the AUTOCITE (automated constraint induced movement extension), Wii games and other robotic devices that can help guarantee increased intensity of practice should also be considered during upper limb rehabilitation. However, the challenges that researchers and clinicians may face in determining whether upper limb or lower limb will recover faster in patients include the role of spontaneous recovery, patients own personal motivation and effort, caregiver support and probably the clinical setting.

## Data availability statement

The original contributions presented in the study are included in the article/supplementary material, further inquiries can be directed to the corresponding author.

## Author contributions

AA, TW, and SN: conception and design, revising it critically for intellectual content, and the final approval of the version to be published. AA: drafting of the paper. All authors contributed to the article and approved the submitted version.

## Funding

This work was supported by the research funding of the Research Centre for Chinese Medicine Innovation of The Hong Kong Polytechnic University (Ref. No. P0041139) awarded to SN and her team; and PolyU Distinguished Postdoctoral Fellowship Scheme (P0035217).

## Conflict of interest

The authors declare that the research was conducted in the absence of any commercial or financial relationships that could be construed as a potential conflict of interest.

## Publisher’s note

All claims expressed in this article are solely those of the authors and do not necessarily represent those of their affiliated organizations, or those of the publisher, the editors and the reviewers. Any product that may be evaluated in this article, or claim that may be made by its manufacturer, is not guaranteed or endorsed by the publisher.
